# Isolation of an aryloxyphenoxy propanoate (AOPP) herbicide-degrading strain *Rhodococcus ruber* JPL-2 and the cloning of a novel carboxylesterase gene (*feh*)

**DOI:** 10.1590/S1517-838246220140208

**Published:** 2015-06-01

**Authors:** Liu Hongming, Lou Xu, Ge Zhaojian, Yang Fan, Chen Dingbin, Zhu Jianchun, Xu Jianhong, Li Shunpeng, Hong Qing

**Affiliations:** 1 Nanjing Agricultural University, College of Life Science, Nanjing Agricultural University, Ministry of Agriculture, Nanjing, China, Key Laboratory of Agricultural Environmental Microbiology, College of Life Science, Nanjing Agricultural University, Ministry of Agriculture, Nanjing, China.; 2 Jiangsu Center for GMO Evaluation and Detection, Jiangsu Academy of Agricultural Sciences, Ministry of Agricultrue, Nanjing, China, Key Lab of Agro-Product Safety Risk Evaluation, Jiangsu Center for GMO Evaluation and Detection, Jiangsu Academy of Agricultural Sciences, Ministry of Agricultrue, Nanjing, China.; 3 Observation and Experimental Station of Saline Land of Coastal Area, Institute of Agricultural Sciences in Coastal Area of Jiangsu, Ministry of Agriculture, Yancheng, China, Observation and Experimental Station of Saline Land of Coastal Area, Institute of Agricultural Sciences in Coastal Area of Jiangsu, Ministry of Agriculture, Yancheng, China.

**Keywords:** fenoxaprop-P-ethyl, microbial degradation, *Rhodococcus ruber* JPL-2, FE -hydrolyzing carboxylesterase gene

## Abstract

The strain JPL-2, capable of degrading fenoxaprop-P-ethyl (FE), was isolated from the soil of a wheat field and identified as *Rhodococcus ruber*. This strain could utilize FE as its sole carbon source and degrade 94.6% of 100 mg L^−1^ FE in 54 h. Strain JPL-2 could also degrade other aryloxyphenoxy propanoate (AOPP) herbicides. The initial step of the degradation pathway is to hydrolyze the carboxylic acid ester bond. A novel esterase gene *feh*, encoding the FE-hydrolyzing carboxylesterase (FeH) responsible for this initial step, was cloned from strain JPL-2. Its molecular mass was approximately 39 kDa, and the catalytic efficiency of FeH followed the order of FE > quizalofop-P-ethyl > clodinafop-propargyl > cyhalofop-butyl > fluazifop-P-butyl > haloxyfop-P-methyl > diclofop-methy, which indicated that the chain length of the alcohol moiety strongly affected the hydrolysis activity of the FeH toward AOPP herbicides.

## Introduction

Fenoxaprop-ethyl{ethyl-2-(4-((6-chloro-2-benzoxazolyl)oxy) phenoxy) propanoate)} (FE) is a representative of aryloxyphenoxy propanoate (AOPP) herbicides. It is used to control a wide range of grasses in wheat fields due to its high target selectivity and low non-target toxicity (Bieringer *et al.*, 1982, Walia *et al.*, 1998). However, research has shown that FE is harmful to aquatic organisms (Asshauer *et al.*, 1990). Consequently, the degradation of its residue is attracting public attention.

In general, while FE and other AOPP analogs in the environment are degraded by both abiotic and biotic processes (Lin *et al.*, 2007, Lin *et al.*, 2008), microorganisms make the largest contribution. AOPP herbicide-degrading microorganisms have been isolated from various genera, including *Sphingomonas*, *Acinetobacter*, *Chryseomonas*, *Pseudomonas*, *Alcaligenes, Rhodococcus*, *Agromyces*, *Stenotrophomonas*, *Aquamicrobium*, and *Microbacterium* (Smith-Grenier and Adkins, 1996a; Smith-Grenier and Adkins, 1996b; Hoagland and Zablotowicz, 1998; Song *et al.*, 2005a; Song *et al.*, 2005b; Hou *et al.*, 2011; Nie *et al.*, 2011; Singh, 2013). The degradation pathway of AOPP herbicides has also been investigated and proposed based on metabolite identification. For example, *Chryseomonas luteola* could degrade diclofop-methyl to diclofop acid and 4-(2,4-dichlorophenoxy) phenol, whereas *Sphingomonas paucimobilis* was capable of degrading diclofop acid to 4-(2,4-dichlorophenoxy) phenol and 2,4-dichlorophenol and (or) phenol (Smith-Grenier and Adkins, 1996a; Smith-Grenier and Adkins, 1996b). In addition, *Pseudomonas fluorescens* converted FE to FA (fenoxaprop acid), and FA was further transformed to CDHB (6-chloro-2,3-dihydrobenzoxazol-2-one) or 2-(4-hydroxyphen-oxy) propionic acid (HPP) in FE-TSB cultures (Hoagland and Zablotowicz, 1998). Hou *et al.* (2011) isolated an FE-degrading bacterium *Rhodococcus* sp. T1, which could hydrolyze FE to FA. CyB (Cyhalofop-butyl)-degrading strain *Pseudomona azotoformans* QDZ-1 could hydrolyze the carboxylic acid ester linkage of AOPP herbicides to their corresponding acid form (Nie *et al.*, 2011). *Pseudomonas* sp. B2 could degrade clodinafop propargyl (CF) to clodinafop acid and 4-(4-Chloro-2-fluoro-phenoxy)-phenol (Singh, 2013). To summarize, the initial step in the degradation of AOPP herbicides, which is shared among these strains, is the breakdown of the carboxylic acid ester bond by a carboxylesterase. Thus far, only two genes encoding this enzyme have been cloned: *chbH* from *Pseudomonas azotoformans* QDZ-1 and *feh* from *Rhodococcus* sp. T1. However, the characteristics of FeH have yet to be investigated.

In this study: JPL-2, an aryloxyphenoxy propanoate (AOPP) herbicide-degrading strain of *Rhodococcus ruber*, was isolated and characterized; a novel carboxylesterase gene *feh* was cloned and expressed; and the characteristics of FeH were studied.

## Materials and Methods

### Chemicals and media

The fenoxaprop-P-ethyl, cyhalofop-butyl, quizalofop-p-ethyl, diclofop-methyl, haloxyfop-p-methyl, fluazifop-p-butyl, clodinafop-propargyl, and fenoxaprop acid were from Langchem Inc. (Shanghai, China). The Luria-Bertani (LB) medium consisted of the following components (in g L^−1^): 10.0 tryptone, 5.0 yeast extract and 10.0 NaCl. Mineral salts medium (MSM) consisted of the following components (in g L^−1^): 1.5 NH_4_NO_3_, 0.5 NaCl, 1.5 K_2_HPO_4_, 0.5 KH_2_PO_4_ and 0.2 MgSO_4_·7H_2_O, pH 7.0. For solid medium, 20.0 g agar was added. The stock solutions of the above herbicides (10000 mg L^−1^, w/v) were prepared in dimethyl sulfoxide and sterilized by membrane filtration. The solutions were added to the sterilized MSM and used as the carbon source when required. Strain JPL-2 could not utilize dimethyl sulfoxide as the sole carbon source for growth in MSM medium.

### Isolation and identification of fenoxaprop-P-ethyl -degrading bacteria

To isolate FE-degrading bacteria, a conventional enrichment method was employed. The soil sample was collected from a wheat field in Henan province that had been subjected to the long-term application of FE. Approximately 2.0 g of the soil sample was added to 100 mL MSM, with the addition of FE (50 mg L^−1^) as the carbon source, and was incubated at 30 °C and 150 rpm for approximately 2 d. Then, 5 mL of the enrichment culture was transferred into another 100 mL of fresh medium for an additional round of enrichment. After three rounds of transfer, the enrichment culture was diluted and spread on MSM agar plates with 50 mg L^−1^ FE. After 2 d of incubation at 30 °C, colonies that degraded FE, as evidenced by the production of a visible transparent halo, were picked and purified. The ability to degrade FE was verified by high performance liquid chromatography (HPLC).

Strain JPL-2 was characterized and identified by morphological, physiological & biochemical characteristics as well as 16S rRNA gene analysis. The morphological, physiological and biochemical characterizations were analyzed according to Bergey's Manual of Determinative Bacteriology (Holt *et al.*, 1994). The strain was also characterized biochemically using the API CORYNE system according to the manufacturer's instructions (API bioMérieux).

Genomic DNA was extracted by the high-salt precipitation method. The 16S rRNA gene of strain JPL-2 was amplified from the genomic DNA with the primers 27F (5′-AGAGTTTGATCCTGGCTCAG-3′) and 1492R (5′-GGTTCCTTGTTACGACTT-3′). The PCR product was purified with a PCR purification kit (Axygen), ligated into the vector pMD18-T (TaKaRa Biotechnology, Dalian, China) and transformed into *E. coli* DH5á. An automatic sequencer (Applied Biosystems, model 3730) was used to determine the 16S rRNA gene sequence. Pairwise sequence similarity was calculated using a global alignment algorithm, implemented by the EzTaxon-e server. Following multiple alignments of the sequence data using CLUSTAL_X (Thompson *et al.*, 1997), phylogenetic analysis was performed using the MEGA version 5.0 software package (Tamura *et al.*, 2011). The G+C content of the genomic DNA was determined by thermal denaturation, with *E.coli* K-12 DNA serving as a reference (Mandel and Marmur, 1968). DNA-DNA hybridizations were performed according to the method of Ezaki *et al.* (1989).

### Growth and degradation experiments

Strain JPL-2 was precultured in LB medium for approximately 12 h to mid-log phase, and the cells were harvested by centrifugation (6,000 rpm for 5 min at room temperature) and washed twice with distilled water. After the cell density had been adjusted to approximately 2.0 at OD_600_, a 1%, (v/v) inoculum was added to 20 mL MSM, with the addition of 100 mg L^−1^ FE, and incubated at 30 °C and 150 rpm. After 6 h, three flasks were taken from the incubator. The bacterial growth was monitored via cell density, and the concentration of FE was determined by HPLC. Each treatment was performed in triplicate, and the control experiments were conducted under the same conditions without inoculation.

Degradation of other AOPP herbicides (clodinafop-propargyl, cyhalofop-butyl, quizalofop-p-ethyl, diclofop-methyl, haloxyfop-p-methyl, and fluazifop-p-butyl) by strain JPL-2 was investigated under the same conditions.

### Cloning of the FE-hydrolyzing carboxylesterase gene (*feh*)

To clone the FE-hydrolyzing carboxylesterase gene, the shotgun method was used to construct a gene library of strain JPL-2 in *E. coli* DH5á. Genomic DNA of strain JPL-2 was extracted by the method described above and digested partially with *Sau*3AI. Fractions containing approximately 4 to 6 kb DNA fragments were pooled, ligated into the *Bam*HI site of the plasmid pUC118 (TaKaRa Biotechnology, Dalian, China), and transformed into competent cells of *E.coli* DH5|Á. The transformants were plated onto LB agar plates containing 100 mg L^−1^ ampicillin and 100 mg L^−1^ FE and incubated at 37 °C for 24 h. Colonies that degraded FE produced clear transparent halos and were screened and further tested for their degrading capabilities. Analysis of the nucleotide and deduced amino acid sequence was performed using Omiga 2.0. BlastN and BlastP were used for the nucleotide sequence and amino acid identity comparison, respectively. (www.ncbi.nlm.nih.gov/Blast).

### Gene expression and purification of FeH

To express the *feh* gene, the open reading frame (ORF) of *feh* lacking a stop codon was amplified by PCR with the primer pair *feh*-F (5′-CTGATTGCATATGTCCGACATCCACGGCGTCT-3′, the *Nde*I site is underlined) and feh-R (5′-ATGCCTCGAGCGGCACGGCGGCGAA-5′, the *Xho*I site is underlined). The amplicon was inserted into the *Nde*I-*Xho*I site of pET29a(+) to generate the recombinant plasmid pET-feh, which was transformed into *E. coli* BL21(DE3). The transformants were subcultured into 100 mL LB medium and allowed to grow until the culture density reached 0.5 (OD_600nm_). To induce the expression of *feh*, isopropyl-β-D-thiogalactopyranoside (IPTG) was added to a final concentration of 1 mmol L^−1^, and the purification of the recombinant *feh* was performed according to the methods of Wang *et al.* (2009). The molecular mass of the denatured protein was determined by sodium dodecyl sulfate-polyacrylamide gel electrophoresis (SDS-PAGE) (Laemmli, 1970), and the protein concentrations were determined by the Bradford method (Bradford, 1976).

### Enzyme assay

The enzymatic activity of FeH and its kinetics toward FE and other AOPP herbicides were determined according to the method of Nie *et al.* (2011) *.* One activity unit was defined as the amount of enzyme required to catalyze the formation of 1 μmol of product per min.

### Chemical analysis

Following culture acidification to a pH 2.0 by the addition of 10% HCl, FE and other AOPP herbicide were extracted from the culture with an equal volume of dichloromethane. The dichloromethane phase was dried over anhydrous Na_2_SO_4_, and the dichloromethane was removed using a stream of nitrogen. The final extract was redissolved in 500 μL methanol and subjected to measurement by HPLC, equipped with a Zorbax C-18 ODS Spherex column (250 mm × 4.6 mm). The mobile phase was 100% methanol, and the flow rate was 1.0 mL min^−1^. Using a 20 μL injection volume, column elutions were monitored by measuring at 237 nm with a Waters 2487 wavelength absorbance detector.

### Identification of the Metabolite of FE Degradation by FeH

The enzyme mixture was added with 100 mg L^−1^ FE and incubated for 30 min in 30 °C. Control experiments with heat-inactivated enzyme were performed under the same conditions. The solution mixture was extracted as described above, and the metabolite was identified by LC-MS (LC-MSD-Trap-SL, Agilent, USA) as described by Liu *et al.* (2012).

### Nucleotide sequence accession numbers

The nucleotide sequences of the 16S rRNA and *feh* genes of strain JPL-2 were deposited in the GenBank database under accession numbers JX110619 and KF601763, respectively.

## Results

### Isolation and identification of the FE-degrading strain

The enrichment procedure obtained a pure culture designated as JPL-2. This strain was gram positive and aerobic. Colonies grown on LB agar were opaque, convex and red. Strain JPL-2 was positive for catalase, hydrolysis of tween-80 and tyrosine, but negative for oxidase, casein, Voges-Proskauer and DNase. It could produce acid from fermentation of D-Fructose, D-Glucose, Glycerol, D-Mannitol and D-Sorbitol but not from L-arabinose, D-cellobiose, D-galactose, inulin, lactose, D-raffinose and L-rhamnose. Growth was observed over a temperature range of 10–42 °C (optimum 30 °C), a salinity range of 0–5% NaCl (optimum 1% NaCl) and a pH range of 4.0–10.0 (optimum 7.0). The 16S rRNA gene sequence of strain JPL-2 showed 100% similarity to *Rhodococcus ruber* DSM 43338^T^(X80625) and 99.48% to *Rhodococcus aetherivorans* 10bc312^T^(AF447391). The DNA G+C content of strain JPL-2 is 66.5 mol%.

**Figure 1:**
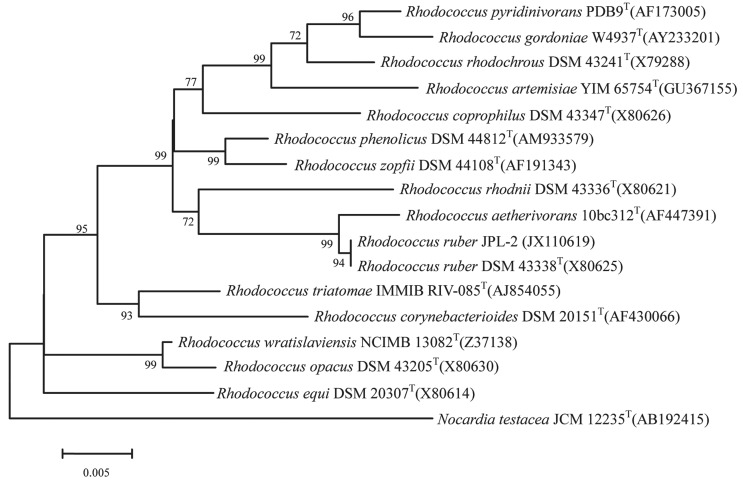
Phylogenetic tree based on the 16S rRNA gene sequences of strain *Rhodococcus ruber* JPL-2 and related species. The scale bar indicates 0.005 substitution per nucleotide position. Bootstrap values obtained with 1000 resamplings are indicated as percentages at all branches.

Strain JPL-2 showed a relatively high DNA-DNA relatedness to strain *Rhodococcus ruber* DSM 43338^T^ (81.6%), which was above the 70% threshold recommended for the delineation of bacterial species (Wayne *et al.*, 1987). Based on the above characteristics, strain JPL-2 was identified as *Rhodococcus ruber* and named *Rhodococcus ruber* JPL-2. A phylogenetic tree based on the known representatives of the *Rhodococcus* species is presented in Figure 1.

### Degradation of FE by strain JPL-2

**Figure 2:**
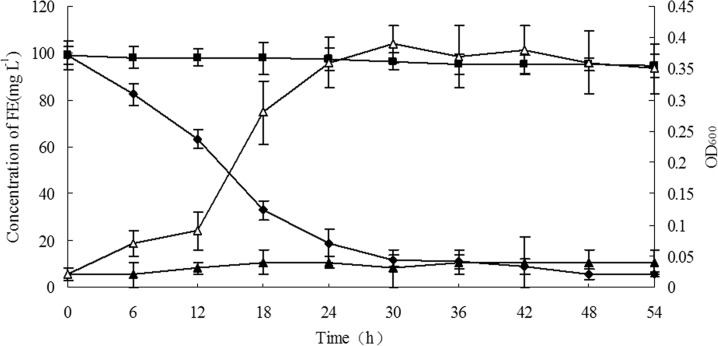
Degradation and utilization of FE during growth of strain JPL-2 in MSM: (n) FE control; (u) degradation of FE; (Δ, ▲) cell Density(OD_600_) of strain JPL-2 with the addition of FE and without addition in MSM medium, respectively.

The growth of strain JPL-2 on liquid MSM supplemented with 100 mg L^−1^ FE and its ability to degrade fenoxaprop-P-ethyl are shown in Figure 2. The growth curve showed a steady increase in the bacterial population. Simultaneously, the HPLC analysis showed a substantial reduction in the concentrations of FE. After incubation for 54 h, approximately 94.6% of the FE was degraded by strain JPL-2. Correspondingly, the OD_600_ increased to 0.35. No significant change in FE concentration was observed in cultures that were not inoculated with strain JPL-2, and no growth was observed for strain JPL-2 when it was inoculated into the culture without the addition of FE. Thus, we concluded that strain JPL-2 was able to degrade and utilize FE as its sole carbon source for growth. For degradation of FE, the optimal temperature and initial pH of the medium were 30 °C and 7.0, respectively (data not shown).

**Figure 3:**
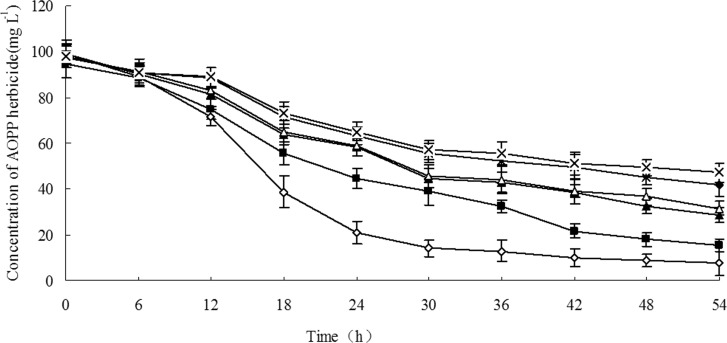
The degradation of other AOPP herbicides by strain JPL-2: (n) Clodinafop-propargyl; (u) Haloxyfop-P-methyl (Δ); Fluazifop-P-butyl (▲); Cyhalofop-butyl (♦); Quizalofop-P-ethyl; (x) Diclofop-methyl.

The degradation capability of strain JPL-2 toward other AOPP herbicides was also investigated. A total of 100 mg L^−1^ of other AOPP herbicides were also used as substrates (Figure 3), and, in 54 h, strain JPL-2 could degrade 83.7% of clodinafop-propargyl, 71.7% of cyhalofop-butyl, 92.4% of quizalofop-p-ethyl, 51.8% of diclofop-methyl, 57.5% of haloxyfop-p-methyl, and 67.7% of fluazifop-p-butyl.

### Cloning and sequence analysis of the *feh* gene

On the plate containing 100 mg L^−1^ FE, a positive clone that produced a transparent halo was screened from approximately 6,000 transformants in the gene library. The inserted fragment in the transformant was 5,428 bp and contained three complete ORFs. The three ORFs were subcloned into the linear vector pMD18-T and transformed into *E. coli* DH5á. One ORF was confirmed to be the target gene encoding the FE-hydrolyzing carboxylesterase. This gene was named *feh*. Sequence analysis indicated that the length of the *feh* gene was 1,140 bp, encoding a protein of 380 amino acids, with a 68.94% G+C content. The *feh* gene showed the highest similarity (78%) with *feh* from *Rhodococcus* sp. T1 (Hou *et al.*, 2011).

### Expression of the *feh* gene in *E. coli* BL21 (DE3)

**Figure 4:**
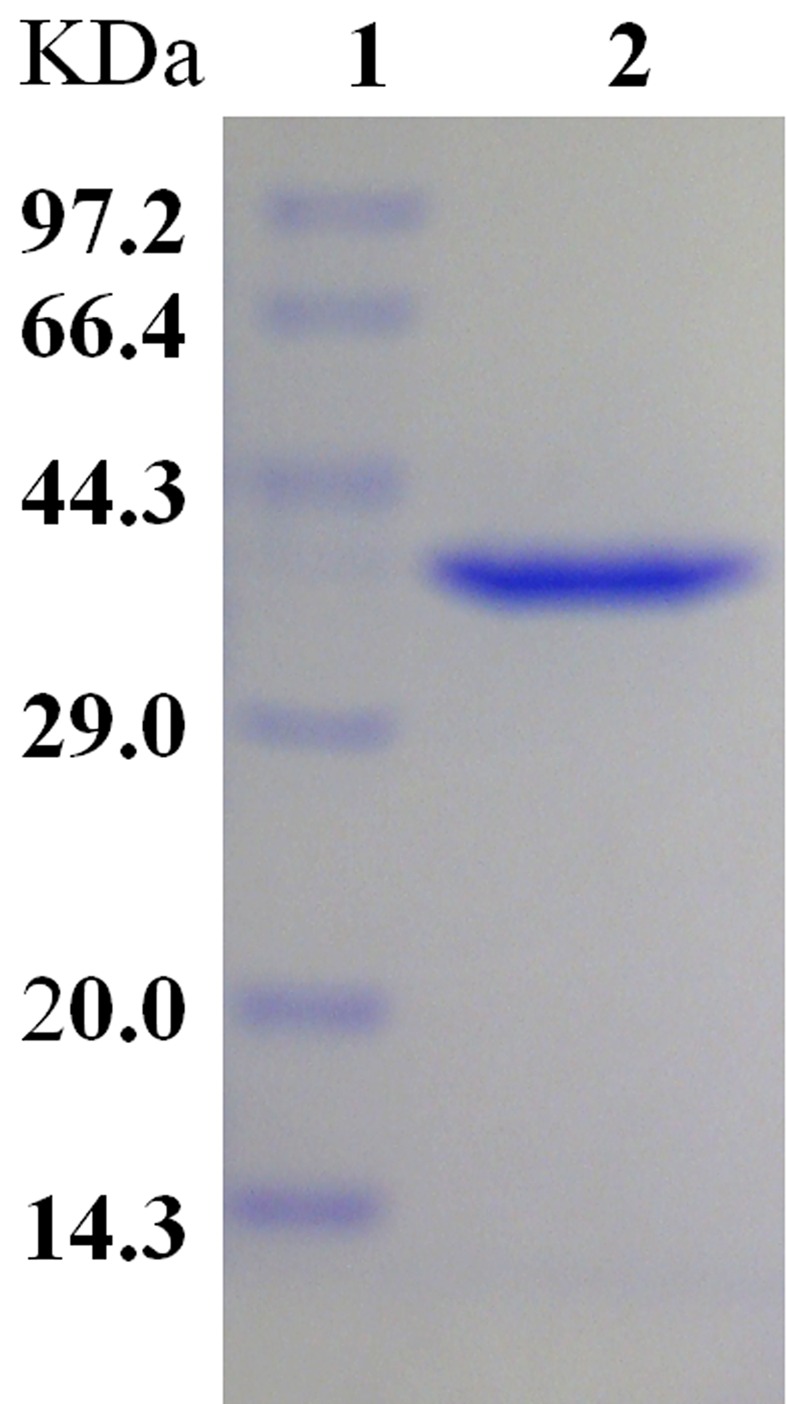
SDS-PAGE analysis of expression of *feh* gene in *Escherichia coli*. Lane 1, protein markers; lane 2, purified FeH.

The FeH produced in *E. coli* BL21(DE3) was purified from crude extract using Ni-nitrilotriacetic acid affinity chromatography, and the purified FeH showed a single band on SDS-PAGE (Figure 4). The molecular mass of the denatured enzyme was approximately 39 kDa, which coincided with the molecular mass calculated from the amino acid sequence.

### Identification of the metabolite

**Figure 5:**
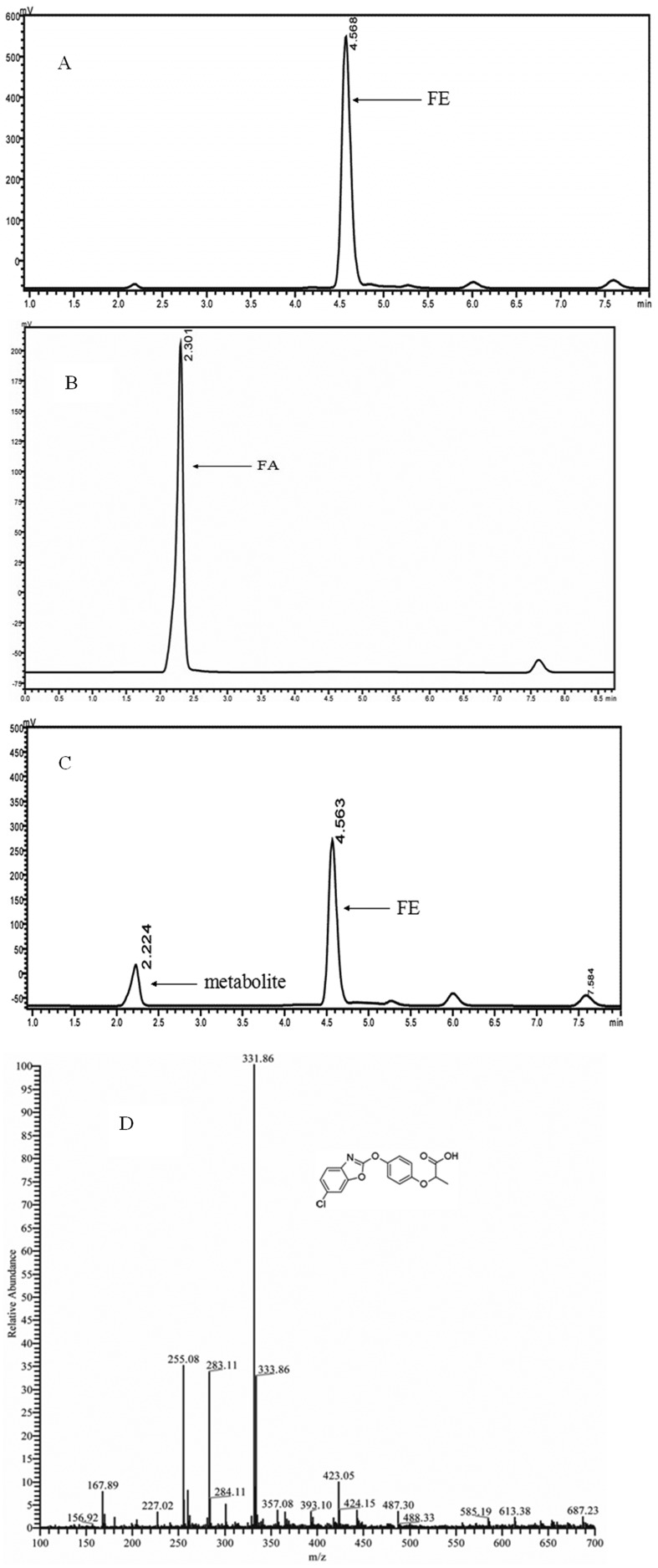
HPLC-MS profile of the metabolite produced by FeH. A, B, HPLC spectra of FE and authentic FA. C, HPLC spectra of FE and its metabolite; D, negatively charged ions mass spectra for metabolite (2.22 min).

When FeH was added to the enzyme reaction mixture, a new metabolite with a retention time of 2.22 min in the HPLC spectrum was produced (Figures 5A and 5C). Its retention time was similar to that of the authentic standards FA (Figure 5B). In the MS spectrum, this metabolite showed a protonated molecular ion at *m/z* 331.9 (Figure 5D), which was consistent with the corresponding mass spectrum of authentic standards FA. Based on these results, we confirmed that FeH catalyzed the hydrolysis of the carboxylic acid ester bond in FE and produced ethanol and FA.

The optimal pH of FeH was observed to be approximately 7.5, but FeH was stable at pH 6.0–8.0. The enzyme was fairly stable up to 45 °C, had 30% residual activity at 60 °C, and was completely inactivated at 70 °C. Many metal ions (Ni^2+^, Hg^2+^ and Ag^+^; 1 mM) as well as the surfactants SDS and Tween80 (10 mM) inhibited enzymatic activity, and the chelating agents EDTA and 1,10-phenanthroline (10 mM) had little effect on the enzyme activity, indicating that FeH had no requirement for metal ions (data not shown).

### Kinetic analysis of FeH

FeH could hydrolyze these AOPP herbicides with different hydrolysis rates in descending order as follows: fenoxaprop-P-ethyl > quizalofop-P-ethyl > clodinafop-propargyl > cyhalofop-butyl > fluazifop-P-butyl > haloxyfop-P-methyl > diclofop-methy (Table 1). This result indicated that the chain length of the alcohol moiety strongly affected the biodegradability of the AOPP herbicides, while the catalytic efficiency of the herbicides with methanol moiety was the lowest. We inferred that the production of methanol would be harmful to the enzyme.

**Table 1:** Kinetic constants of FeH towards different AOPP herbicides.

	Specific activity (mmol/min/mg)	*k* *_cat_* (s^−1^)	*K* *_m_* (mM)	*k* *_cat_* */K* *_m_* (mM^−1^ . s^−1^)
fenoxaprop-P-ethyl	1.08	0.70	0.20	3.52
quizalofop-P-ethyl	1.16	0.75	0.23	3.25
clodinafop-propargyl	0.94	0.61	0.29	2.09
cyhalofop-butyl	0.92	0.6	0.32	1.85
fluazifop-P-butyl	0.90	0.59	0.36	1.65
haloxyfop-P-methyl	0.57	0.37	0.39	0.94
diclofop-methyl	0.51	0.33	0.41	0.81

## Discussion

In the present study, an AOPP-degrading strain JPL-2 was isolated from a wheat field subjected to the long-term application of FE in the Henan province of China. We identified the strain as *Rhodococcus ruber*. Some bacteria capable of degrading AOPP herbicides have previously been isolated. For example, *Alcaligenes* sp. H could degrade 45.8% of 100 mg L^−1^ fenoxaprop-p-ethyl within 5 d (Song *et al.*, 2005a). *Pseudomonas fluorescens* strains RA-2 and UA5-40 cultured in tryptic soy broth (TSB) completely hydrolyzed FE to FA within 5 d (Hoagland and Zablotowicz, 1998). Six nonfermentative Gram-negative bacilli utilized 1.5 mg L^−l^ diclofop-methyl after 31 h of incubation at 25 °C. *Chryseomonas luteola* completely degraded 1.5 mg L^−l^ diclofop-methyl to diclofop acid and 4-(2,4-dichlorophenoxy)phenol within 71 h, and *Sphingomonas paucimobilis* could mineralize 1.5 mg L^−l^ diclofop-methyl to diclofop acid in 54 h (Smith-Grenier and Adkins, 1996a; Smith-Grenier and Adkins, 1996b). *Pseudomonas* sp. Strain B2 was able to degrade 87.14% of 80 mg L^−l^ clodinafop propargyl in 9 h (Singh, 2013). *Rhodococcus* sp. T1 could degrade 94% of 100 mg L^−l^ FE within 24 h (Hou *et al.*, 2011). *Pseudomonas azotoformans* QDZ-1 degraded 84.5% of 100 mg L^− l^ cyhalofop-butyl after 5 d of incubation (Nie *et al.*, 2011). Strain JPL-2 could degrade 94.6% of 100 mg L^−1^ FE within 54 h and could also use other AOPP herbicides like cyhalofop-butyl, quizalofop-p-ethyl, diclofop-methyl, haloxyfop-p-methyl, fluazifop-p-butyl and clodinafop-propargyl as substrates. In comparison with the reported strains, strain JPL-2 is not only capable of rapid FE degradation but it is also a broad substrate degrader.

In general, the initial step of the metabolism of AOPP herbicides involves the breakdown of the carboxylic acid ester bond. Strain JPL-2 hydrolyzed FE to FA and ethanol, although the latter was not detectable by HPLC chromatography because ethanol cannot absorb UV light. Ethanol could also be further used by strain JPL-2 (data not show). Currently, two genes encoding carboxylesterase have been cloned: chbH, from *Pseudomonas azotoformans* QDZ-1 (Nie *et al.*, 2011), which was also able to hydrolyze the AOPP herbicides, and *feh*. The *feh* gene was first cloned from *Rhodococcus* sp. T1 (Hou *et al.*, 2011), but the characteristics of FeH were not extensively investigated. In the present study, another *feh* gene was cloned from strain JPL-2, and the characteristics of FeH were studied in detail. FeH from strain JPL-2 has 78% similarity to that from *Rhodococcus* sp. T1, but it only exhibited a 10% similarity to ChbH. The Vmax of FeH was approximately 300 times that of ChbH toward the substrate FE, and it has a broader substrate spectrum. However, FeH and ChbH share a common characteristic: against the tested substrates, their enzymatic activity decreased as the length of the aliphatic chain flanking the ester bond of the AOPP herbicides increased.

## Conclusion

Strain JPL-2 was isolated from the soil of a wheat field and identified as *Rhodococcus ruber*. A novel carboxylesterase gene (*feh*), enabling the strain to efficiently degrade AOPP herbicides, was cloned from JPL-2. FeH from strain JPL-2 has a 78% similarity to that from Rhodococcus sp. T1, but it only showed a 10% similarity to ChbH. FeH had a broader substrate spectrum and higher catalysis efficiency than ChbH.
